# Image-based biomechanical models of the musculoskeletal system

**DOI:** 10.1186/s41747-020-00172-3

**Published:** 2020-08-13

**Authors:** Fabio Galbusera, Andrea Cina, Matteo Panico, Domenico Albano, Carmelo Messina

**Affiliations:** 1 IRCCS Istituto Ortopedico Galeazzi, Milan, Italy; 2 Department of Chemistry, Materials and Chemical Engineering “Giulio Natta”, Politecnico di Milano, Milan, Italy; 3 Department of Biomedicine, Neuroscience and Advanced Diagnostics, Università degli Studi di Palermo, Palermo, Italy; 4 Department of Biomedical Sciences for Health, Università degli Studi di Milano, Milan, Italy

**Keywords:** Artificial intelligence, Finite element analysis, Musculoskeletal System, Tomography, Tomography (x-ray computed), Magnetic resonance imaging

## Abstract

Finite element modeling is a precious tool for the investigation of the biomechanics of the musculoskeletal system. A key element for the development of anatomically accurate, state-of-the art finite element models is medical imaging. Indeed, the workflow for the generation of a finite element model includes steps which require the availability of medical images of the subject of interest: segmentation, which is the assignment of each voxel of the images to a specific material such as bone and cartilage, allowing for a three-dimensional reconstruction of the anatomy; *meshing*, which is the creation of the computational mesh necessary for the approximation of the equations describing the physics of the problem; *assignment of the material properties to the various parts of the model*, which can be estimated for example from quantitative computed tomography for the bone tissue and with other techniques (elastography, T1rho, and T2 mapping from magnetic resonance imaging) for soft tissues. This paper presents a brief overview of the techniques used for image segmentation, meshing, and assessing the mechanical properties of biological tissues, with focus on finite element models of the musculoskeletal system. Both consolidated methods and recent advances such as those based on artificial intelligence are described.

## Key points

- Finite element analysis allows predicting quantities not measurable *in vivo* or *in vitro*.
- Medical imaging plays a critical role in state-of-the-art finite element models.
- Patient-specific finite element models are typically based on computed tomography and magnetic resonance imaging.
- Mechanical properties of biological tissues can be estimated by applying finite element analysis to three-dimensional imaging techniques.

## Background

In the last decades, finite element analysis (FEA) has become a precious tool for the investigation of the biomechanics of the musculoskeletal system. In comparison with other methods such as *in vivo* measurements (*e.g.,* imaging, motion analysis) and *in vitro* testing of cadaveric specimens, FEA allows for the prediction of quantities which are not measurable by means of experimental techniques, such as local stresses and strains, do not require large investments such as those necessary for acquiring and managing cadaver specimens, complex testing infrastructures, or motion analysis laboratories [1, 2].

**Fig. 1:**
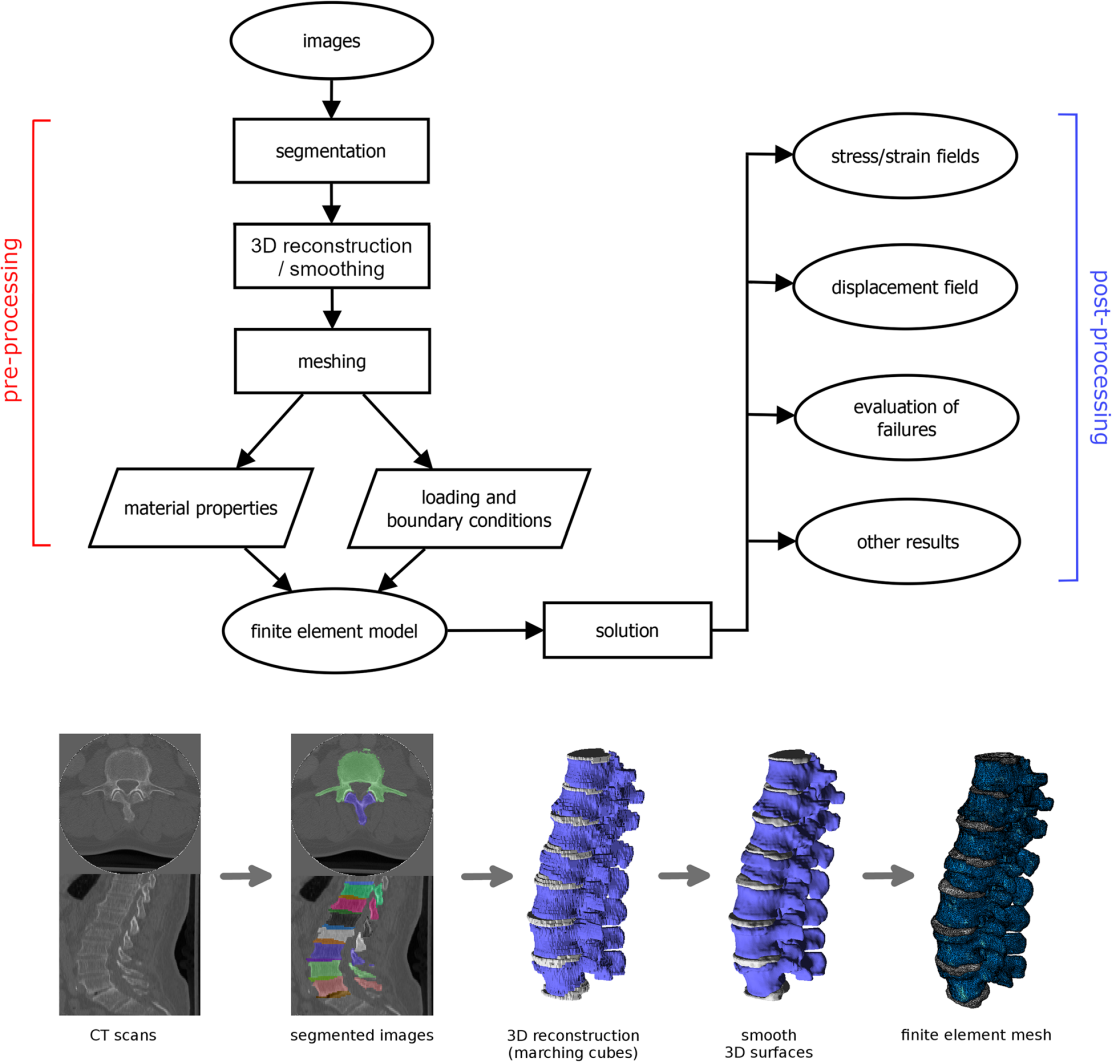
The workflow for the development and use of a finite element model from medical images (for example computed tomography scans): segmentation, three-dimensional reconstruction, improvement of the quality of the reconstructed surfaces by means of filtering (smoothing), meshing, assignment of loading/boundary conditions, and of material properties. The model can then be used to make predictions about stresses, strains, displacements, evaluating the failure of the materials, etc. Partially reprinted with permission from [1]

Medical imaging plays a critical role in the development of anatomically realistic, state-of-the-art finite element models to be used for biomechanical investigations. Indeed, the workflow for the model development starts from medical images of the subject of interest, typically computed tomography (CT) or magnetic resonance imaging (MRI) scans, which are then used in the successive steps for the construction of the model:

1. Segmentation, *i.e.,* the assignment of each pixel/voxel of the images to a specific material such as bone, and cartilage;
2. Two-dimensional (2D) or three-dimensional (3D) reconstruction of the geometry of the various components of the model, possibly followed by the application of enhancing filters (*e.g.,* surface smoothing);
3. Meshing, *i.e.,* the creation of the computational mesh, or grid, necessary for the approximation of the equations of mechanics [3, 4];
4. Assignment of the material properties to the various parts of the model (Fig. 1) [1].

The finite element model is then ready to be used for the simulation of any biomechanical scenario, through the application of appropriate boundary and loading conditions.

Although several papers described in detail the individual steps of the development of a finite element model, the available literature does not include a review paper focusing on the use of medical images for the generation of an anatomically accurate model, especially considering recent advances such as artificial intelligence techniques and state-of-the-art methods for extracting material properties from the images themselves. This narrative review therefore aims at analysing the steps of the workflow for the development of a finite element model which are based on medical imaging, namely, segmentation, meshing, and patient-specific assessment of the material properties of the tissues, highlighting recent improvements and future perspectives.

## Segmentation

Segmentation is the process of partitioning an image into multiple segments that contain pixels with similar features [5]. In medical imaging, these different segments are usually related to different tissue classes, for example, muscles, fat, or bone tissues. The main goal of segmentation in the medical domain is to define object boundaries in images to create 3D reconstructions of the body segment of interest, which is the first step toward the generation of a finite element model. Moreover, segmentation can be used for the study of tissue diseases such as osteoarthritis, for example, by extracting important geometrical features from the 3D shape and size of the joint that are useful for studying the pathology [6].

From a technical point of view, segmentation techniques can be divided in methods based on thresholding, edge detection, snakes, regions, clustering, watershed, partial differential equations, and artificial neural networks (ANN) [7]. Whereas some of these techniques require the intervention of a human user, others are able to perform the task of segmentation automatically. Threshold-based methods subdivide the images with respect to pixel intensities; threshold values could be either manually or automatically selected. In edge- and snakes-based methods, the aim is detecting directly the object boundaries in order to segment the regions of interest. Region-based methods segment the images starting from a seed (initial pixels), selected either manually or automatically, that grows step by step forming a region of interest. Segmentation methods based on clustering techniques aim at dividing the image into clusters of pixels with similar features. Watershed methods use the gradient of image (in terms of difference between pixel values) to determine the boundaries of each region, which correspond to the pixels having the highest gradients. Partial differential equations are used mainly to detect edges and boundaries, then employed to build the actual segments in the image. Finally, ANNs are currently the golden standard for medical images segmentation thanks to their state-of-the-art performances in the detection and identification of regions of interest.

Detailed technical descriptions of the segmentation methods described above are widely available elsewhere [7, 8]. The next paragraphs present a brief overview of medical image segmentation paying specific attention to the methods which have been used in the musculoskeletal area for the creation of image-based biomechanical models.

### Manual and semiautomatic segmentation

**Fig. 2:**
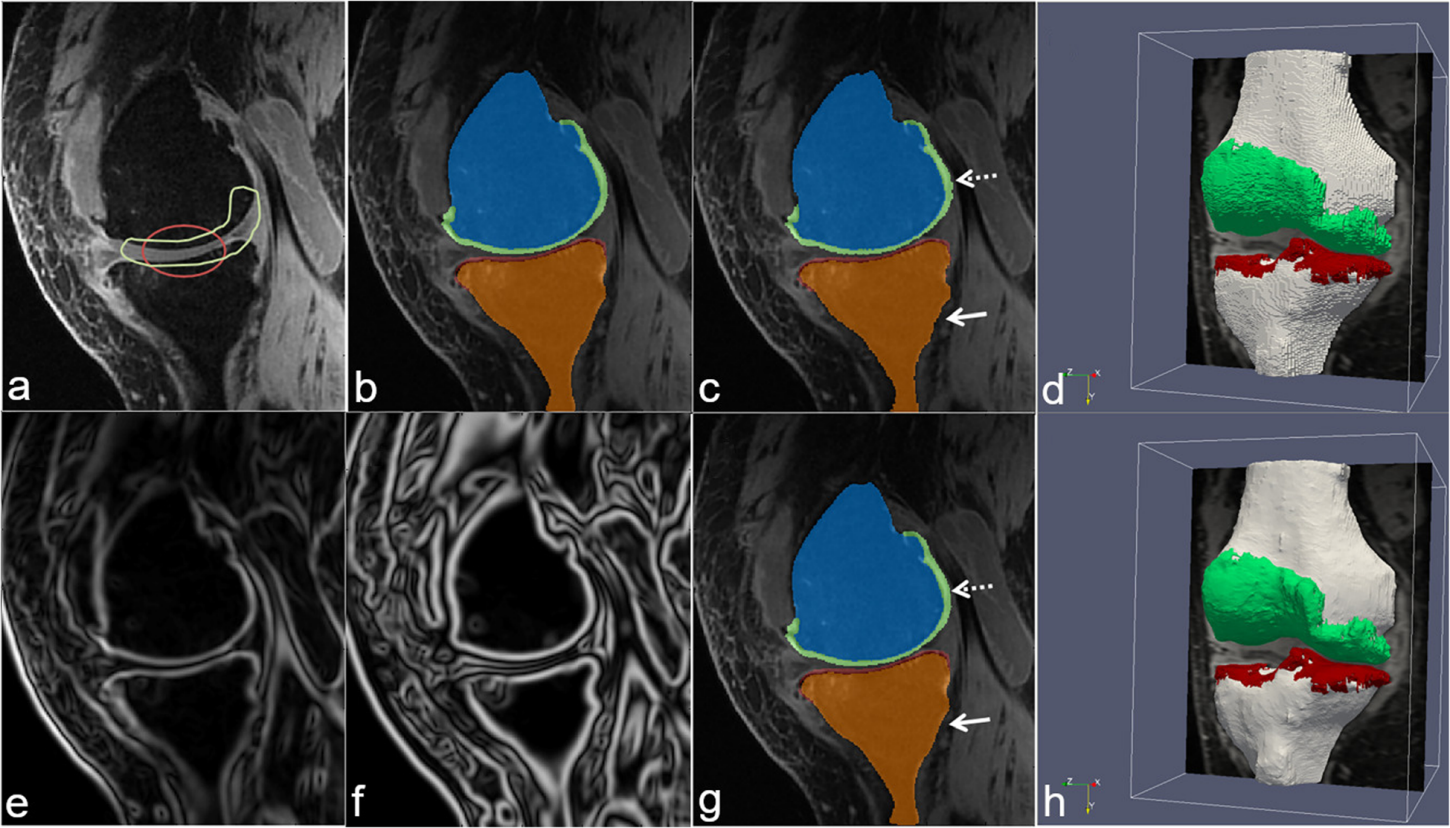
Example of segmentation of a knee joint from magnetic resonance imaging scans, manually performed by a human operator, automatically determined by a state-of-the-art deep learning method or with a novel method employing neural networks and a deformable model described in [10]. **a** Definition of the region of interests for femoral cartilage (green) and tibial cartilage (red); **b** manual segmentation created by an expert human operator; **c** automated segmentation obtained with a state-of-the-art deep learning method; **d** three-dimensional reconstruction of the automated segmentation; **e**, **f** filtered images enhancing the contours necessary for the novel approach; **g**, **h** segmented slice and three-dimensional reconstruction of the segmentation obtained with the novel method. Reprinted with permission from [10]

Manual segmentation is a process in which an expert transcriber identifies different zones on an image and assigns them to a specific category. Manual segmentation is considered the most accurate technique if properly performed, but it is time-consuming and it is affected by inter- and intra-user variability [9] (Fig. 2).

Project-specific tools are commonly developed to support manual segmentation. For example, a group of researchers developed a tool for subject-specific femur modeling [11]. The graphical user interface allows the user to interact with the image stack through a 3D scene and a 2D scene. The main segmentation tools are a brush which adds single segmentation points, and a curve tool which uses splines to add a connected sequence of segmentation points.

To overcome the limitations of manual segmentation, some tools to enable a semiautomatic procedure have been developed. Such tools combine the robustness of manual segmentation with the speed of automatic segmentation and are therefore widely used. As mentioned above, semiautomatic methods can be based on thresholding, region growing, edge detection, snakes, etc. In all these methods, the user must input and set some parameters interactively [12]. The supposed advantages of the semiautomatic approach over manual segmentation was demonstrated by Scheys et al. [13], who developed a pipeline to build subject-specific musculoskeletal models allowing for a quantitative comparison of the two techniques; a 50% time gain for the semiautomatic method compared to the manual one was demonstrated.

A research group presented a tool for the semiautomatic segmentation of MRI spinal cord images [14], in which the user had to mark the centreline of the spinal cord (which acts as seed) on a few slices, which was then used by an automatic segmentation algorithm to perform the segmentation of the cord itself. The cross-sectional areas calculated by the segmentation were found to be associated with relevant clinical disability scores.

Another group of researchers used a semiautomatic approach for segmenting images of the shoulder [15]. The segmentation process exploited an established system of placing seed points representing the object of interest and other points representing the background. The developed tool was able to perform the segmentation of the humerus, scapula, and clavicle, and to use them as reference to create a statistical representation of the position of the deltoid muscle.

Stammberger et al. [16] made a comparison between B-Spline snakes and manual segmentation in the measurement of the thickness of the articular cartilage. The developed semiautomatic algorithm was able to delineate the cartilage boundaries with minimal manual interventions. The semiautomatic segmentation showed excellent intra-observer repeatability in the femoral and tibial cartilage volumes (intraclass correlation coefficient 0.989 and 0.965, and mean differences of 1.9% and 3.0% for repeated segmentations, respectively).

Cartilage segmentation was also the aim of a study by Liukkonen et al. [17] in which a radial intensity-based method was applied. The user had to mark a reference point from the central point of bone and edge points of the cartilage from a sagittal slice of the knee. Then, an automatic algorithm calculated intensity profiles of the pixel to obtain the segmentation.

### Automatic segmentation

In automatic segmentation, the thresholds or the boundaries of the objects of interest are automatically assigned by a program without human intervention [18]. These approaches can allow for a significant reduction of required time and human resources, at the risk of possibly imprecise results due to the complex morphology and anatomy of the musculoskeletal system [19].

#### Automatic thresholding

The concept of automatic thresholding is to automatically select one or more optimal grey-level threshold values to separate objects of interest in an image [11]. One of the best performing methods is the Otsu technique [20], which outperformed other sophisticated techniques that used the entropy of the histogram of the pixel values [21, 22] or minimum error methods [23]. The method was further improved by [11], who addressed its sensitivity to object size. Indeed, in the original formulation of Otsu thresholding, objects larger than the background may be classified as background and vice versa.

#### Artificial intelligence

**Fig. 3:**
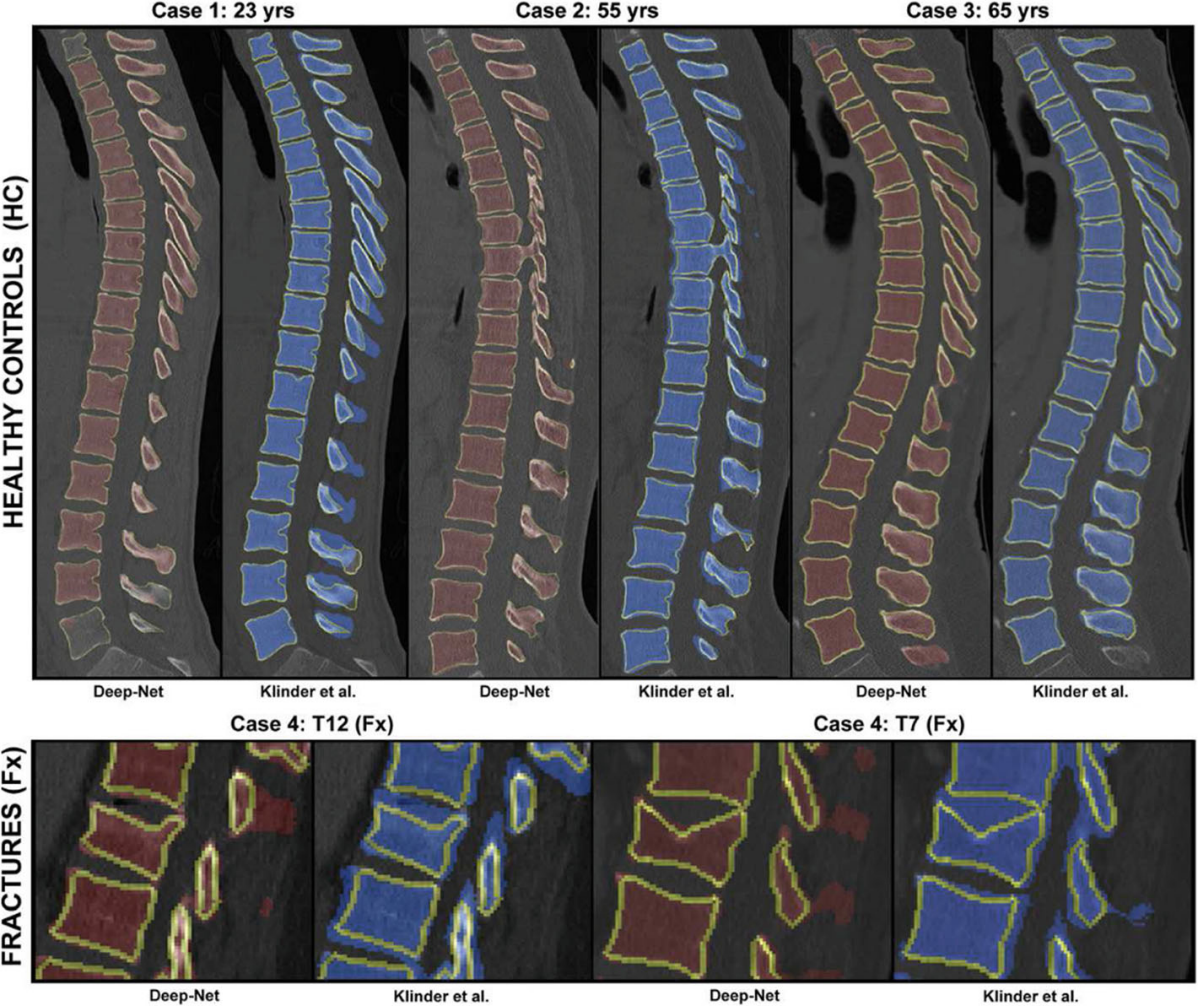
Comparison of the performance of a tool based on a state-of-the-art deep learning architecture (Deep-Net) [24] and a more conventional, model-based method [25] for the segmentation of computed tomography scans of healthy and pathological spines, highlighting the improved performance of the deep network especially for the pathological cases. Reprinted with permission from [24]

In the last years, artificial intelligence (AI) and in particular deep learning have been widely used in computer vision tasks, among which for the segmentation of images, thanks to their state-of-the-art performances which are currently unmatched by the more traditional image processing methods (Figs. 2 and 3). The boost in performance was facilitated by the development of deep ANN architectures, especially convolutional neural networks (CNNs), and by the availability of the large datasets of segmented images.

In the musculoskeletal field, a group of researchers used a CNN to detect the edge in ultrasound images [26]. The authors trained the network to recognise if a pixel is an edge or non-edge pixel using a ground truth obtained both from a Canny edge detector [27] and manual annotations by an expert anatomist. The performances were evaluated using several metrics and highlighted very good performances.

U-Net is a convolutional network architecture widely used for fast and precise segmentation of images. In a work by Hiasa et al. [28], a Bayesian convolutional network with the U-Net architecture was implemented to automatically extract muscle segmentation from clinical CT scans. The authors evaluated the performances on two datasets using the Dice coefficient (DC) [29] and the average symmetric surface distance (ASD). The results show a DC of 0.89 and an ASD of 0.994 mm, which constituted a statistically significant improvement in comparison with other state-of-the-art techniques such a multi-atlas method and another ANN-based approach, the FCN-8s architecture [30].

Concerning segmentation of MRI images, Liu et al. combined a deep CNN with a 3D deformable model with the aim of improving the segmentation accuracy of cartilage and bone within the knee joint [10] (Fig. 2). The authors used a SegNet architecture to perform pixel-wise tissue classification, and they achieved good performances in terms of accuracy and computational costs.

Recent advances in deep learning demonstrated the capabilities of generative models, which could learn complex distributions in images, in image segmentation tasks, and in particular for the detection of abnormalities. Among the various solutions based on generative models, Chen et al. recently showed the potential of using variational and adversarial auto-encoders to detect abnormalities in medical images [31].

## Meshing

The segmented image is then used to construct an accurate 3D model of the anatomy and to subdivide it into smaller domains called elements [32]. This process is called finite element meshing and is essential for developing biomechanical models for FEA; indeed, a good-quality mesh is necessary to obtain accurate results from the simulations. Besides, the choice of the meshing techniques may have a critical impact on the degree of anatomical and geometrical fidelity of the resulting biomechanical model [33, 34].

**Fig. 4:**
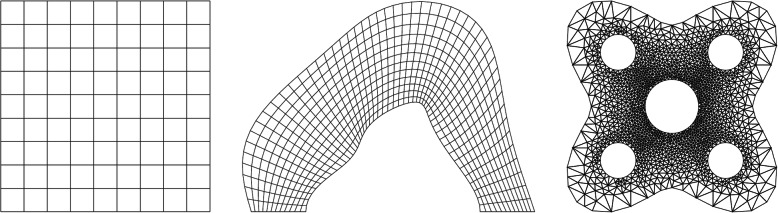
Examples of structured mesh on a regular domain (left), structured mesh on an irregular domain (middle) and unstructured mesh on an irregular domain (right). Reprinted with permission from [35]

Finite element meshes can be classified as either structured or unstructured depending on the local grid connectivity (Fig. 4). Structured meshes have a regular pattern of connections between elements. Structured grids cannot always be used to create high-quality, anatomically realistic meshes, depending on the geometrical complexity of the domains; in such cases, unstructured meshes should be preferred. Indeed, although special techniques to adapt unstructured grids to complex geometries are available, in some cases, they lack the necessary flexibility to create suitable meshes in the near-wall regions. In general, although structured meshes are traditionally preferred due to their inherent higher accuracy, unstructured meshes consisting of higher order elements can provide acceptable accuracy and convergence characteristics [35, 36]. Hybrid structured/unstructured grids able to combine the capability of unstructured meshing to resolve complex geometries and the high accuracy of structured meshing in the regions far from the boundaries are also being increasingly employed [35, 37].

### Unstructured meshing

Unstructured meshes are most commonly composed of triangular (2D) or tetrahedral (3D) elements. The techniques used to create unstructured meshes can be classified as Delaunay-based, advancing-front base, and octree-based methods [35]. Delaunay-based methods generate and refine the Delaunay triangulation of a set of progressively inserted vertices inside/on a given surface domain. This technique is very effective in 2D mesh generation but may not work well in generating tetrahedral meshes, in which a number of nearly degenerate elements, commonly known as slivers, often occur. Advancing front-based methods start from the boundary of a domain and then insert new points inside it in order to generate triangles or tetrahedra with acceptable shapes and desired size. These methods are sensitive to the quality of the surfaces describing the boundaries, which typically consists of triangles in the 3D space; poorly shaped boundaries often give rise to a low-quality mesh. Octree-based methods recursively subdivide the domain until some predefined stopping criteria are reached. Other methods aimed at increasing the quality of a tetrahedral mesh exist: topology optimisation which modifies the connectivity between mesh vertices while keeping vertex positions unchanged; vertex insertion/deletion inside the mesh; vertex smoothing, which repositions the coordinates of the vertices while keeping the connectivity unchanged [38, 39]; variational methods, which account for the intensity of the underlying image to optimise the position of nodes [40, 41].

### Structured meshing

A structured mesh usually comprises entirely quadrilateral (2D) or hexahedral (3D) elements. The structured meshing of an object enclosed by a single surface consists of the following steps: (1) a set of points on the surface of the object and a few interior points are obtained; (2) an implicit function is constructed such that the function defines the surface of the body; (3) a set of voxels for 3D (pixels for 2D) that encompass the entire domain for which the implicit function are constructed; (4) a finite element discretisation is obtained based on the voxels that are encompassed by the implicit surface. The selection of the voxel size is a crucial step in this procedure, since it is associated with both the accuracy of the finite element solution and with the computational resources necessary to solve the finite element problem.

A special case of structures meshing is the voxel-based finite element meshing method, which directly converts the segmented images from 3D voxels to eight-node hexahedral elements. This method is unable to capture complex domains, which may in turn lead to inaccurate finite element analysis results near the boundaries, especially in case of a large voxel size. Voxel-based meshes are however commonly employed for computational biomechanical investigations, especially for the study of the biomechanics of trabecular bone, due to the ability of voxel-based modeling to automatically assign properties based on greyscale intensities [42, 43].

An alternative approach for the creation of structured meshes is based on statistical shape modeling and mesh morphing, in which an existing high-quality mesh is fitted to a patient-specific anatomy [44]. These techniques allow for the semiautomatic generation of anatomically realistic structured meshes in a relatively short time and are thus gaining popularity for patient-specific modeling [45].

### Combining unstructured and structured mesh

Hybrid meshes consisting of both hexahedral and tetrahedral elements can be created by using the marching-cubes algorithm, which defines elements based on the value (inside the domain, outside of it, on the boundary) of the eight voxels which constitute a cube, the basic shape of a hybrid mesh [46, 47]. When a voxel corresponding to one of the eight vertices of the cube is considered to be on the boundary, a triangular facet is created dividing the cube into two portions in order to create tetrahedral elements. In this way, the outer layer of the hybrid mesh is composed of tetrahedral elements, while the internal region of hexahedral elements. The two layers share the same set of nodes and are connected seamlessly, thus maintaining finite element compliance. This method allows the assignment of heterogeneous mechanical properties to different structures. This meshing technique allows for a good performance in terms of both geometry conformability and finite element accuracy [38, 43].

### Meshing of musculoskeletal models

**Fig. 5:**
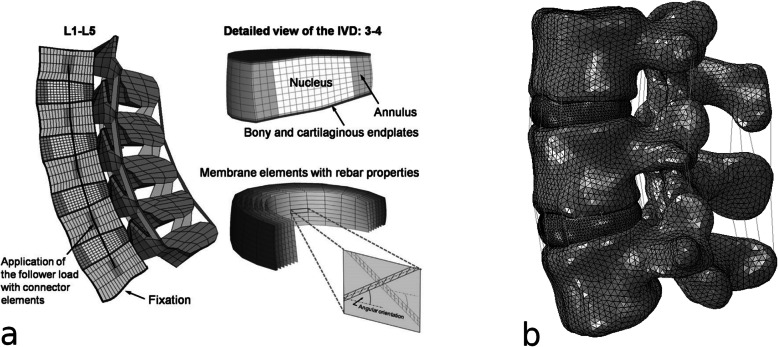
Examples of meshes of a model of the lumbar spine: **a** structured mesh, highlighting the special techniques used to model the collagen fibre-reinforced nature of the intervertebral disc; **b** unstructured mesh. **a** Reprinted with permission from [48]

Musculoskeletal models meshed with both tetrahedral elements or hexahedral elements have been described (Fig. 5). In general, tetrahedral elements are used most frequently since this element type can conform to the complex boundaries of anatomical structures; however, higher-order tetrahedral elements or high mesh density, resulting in higher computational costs, is often required to achieve good accuracy. Hexahedral elements are therefore favoured whenever possible. As mentioned above, geometrically complex anatomical structures cannot always be decomposed into an assembly of hexahedral elements, and hybrid meshes are therefore being increasingly used [38, 43].

## Identification of material properties from imaging

The development of a finite element model requires the assignment of material properties, such as elastic modulus, Poisson ratio, and tensile strength, to each of its elements. Whereas most published models do not attempt to replicate in detail the local properties of the tissues on a patient-specific basis, in several papers information about the material properties is directly extracted from the images and then implemented in an element-wise fashion in the models. Such an approach has been widely employed for the simulation of the local properties of bone based on quantitative CT (QCT) and high-resolution QCT (HR-QCT) [49, 50], using various techniques to assign the properties to either nodes or elements [51, 52]. Additionally, other methods based on MR and ultrasound elastography [53] as well as special MR techniques such as delayed gadolinium-enhanced MRI of cartilage (dGEMRIC), T1rho and T2-mapping [54] have also been used for the creation of numerical models.

### Bone properties from QCT and high-resolution CT

Dual energy x-ray absorptiometry (DXA) is the most widely used technique for quantitative bone assessment in clinical practice. Despite this, DXA is not able to assess volumetric bone mineral density (BMD) or bone geometrical parameters [55], which make it less suitable for estimating material properties for finite element modeling. Thus, alternative techniques have been explored to assess bone properties, such as the use of QCT and HR-QCT. The typical aim of such models is improving fracture risk prediction by simulating mechanical loads applied to models with image-derived material properties (strength, stiffness, etc.) [56].

A QCT-based FEA study showed that the mechanical properties are associated with fracture in all loading conditions [57]. Interestingly, the study showed that the strength calculated with FEA in the posterolateral loading in men or the posterior loading in women was more associated with incident hip fracture [57]. Similarly, another study confirmed the association between QCT-based FEA measures of vertebral strength and incident vertebral fracture, with similar or better ability in fracture prediction compared to areal BMD [58]. Of note, a FEA study on standard CT of vertebral cadaveric specimens showed strong linear correlation between CT/FEA bone strength and the results of the experimental tests (*r* = 0.938, *p* < 0.0001) [59]. Finite element models based on CT have also been successfully employed for the study of load transfer in the cranio-maxillofacial skeleton [60].

Since voxel size was shown to impact the accuracy of FEA results [61], high-resolution imaging has the potential to markedly improve the quality of the predictions. HR-QCT have been used to perform 3D measurements of the peripheral skeleton (typically radius and tibia) to get information about volumetric BMD, cortical/trabecular geometry, and ultimately on bone quality. These finite element models with material properties derived from HR-QCT are typically built by directly transforming the voxels into elements [56]. Cadaver studies at distal radius and tibia showed very high correlation between FEA-derived parameters of bone strength and stiffness with loading parameters (with *r*^2^ > 0.9) [62, 63]. Clinical studies on patients with forearm fractures showed that FEA-HR-QCT quantities, such as FEA-estimated failure load, were poorer at contralateral radius compared to control subjects without fractures [64]. Finally, studies have shown a good and statistically significant correlation between HR-QCT FEA observations and those measured with QCT at both central sites, suggesting a possible use of peripheral measurement as surrogate for getting information from hip and lumbar spine [65]. As a matter of fact, it has to be noted that the use of HR-QCT is still limited in clinical practice due to reduced availability of devices and the higher costs compared to DXA. Thus, results from HR-QCT-derived finite element modes are still considered an exquisite research tool, despite the very promising results in terms of fracture prediction [56].

### MRI/ultrasound elastography

**Fig. 6:**
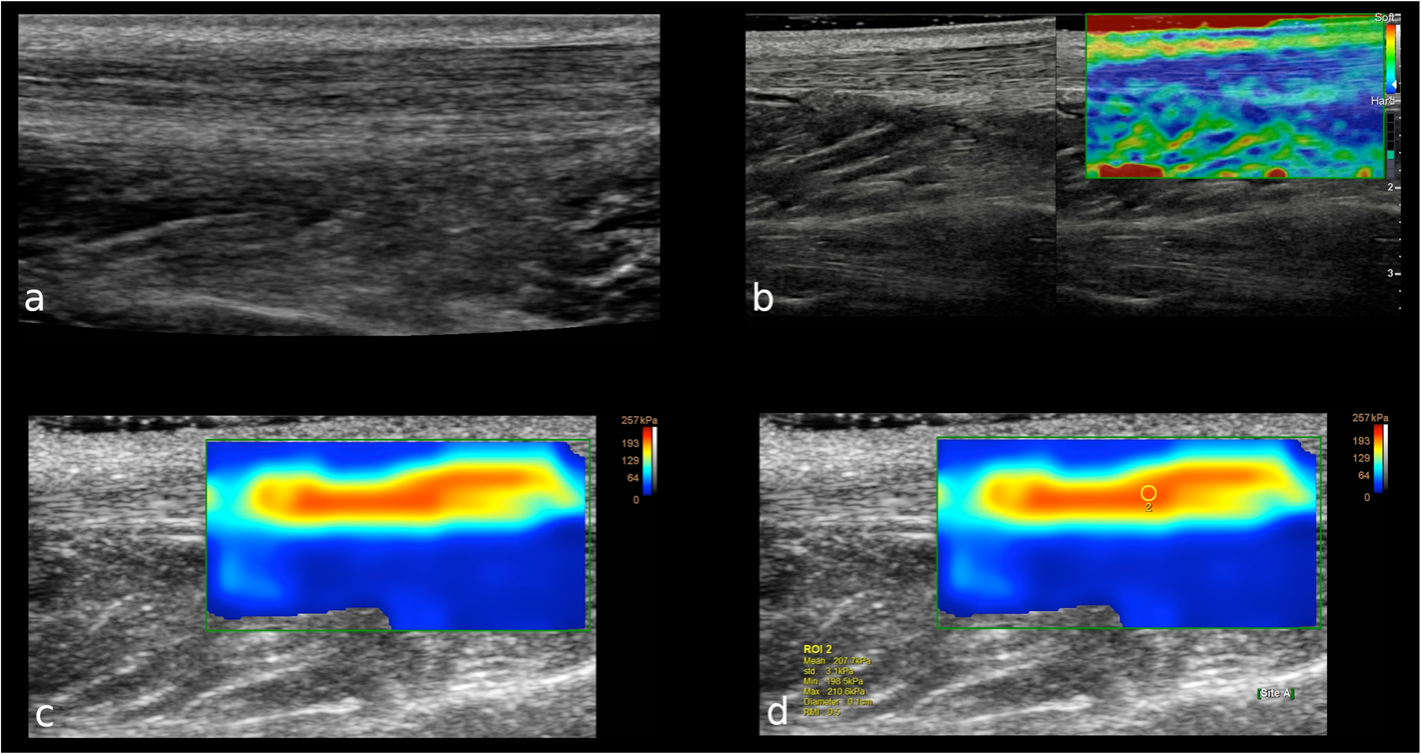
A 31-year-old male patient with healthy left Achilles tendon. **a** B-mode ultrasound image on longitudinal axis shows the normal thickness and echotexture of the proximal third of the Achilles tendon. **b** Longitudinal real-time strain sonoelastography shows the normal appearance of the proximal third of the Achilles tendon as blue, which represents stiff tissue. Subcutaneous fat tissue over the tendon appears yellow to green indicating soft tissue. **c** Shear wave elastography shows that the normal tendon is hard (red) and homogeneous, with the softer tissue over and below the tendon that is easy to distinguish. (**d**) The box is the region of interest to calculate the tendon elasticity

Elastography is an imaging tool able to map tissue stiffness investigating the response of biologic tissues to an excitation and tracking propagating strain waves (Fig. 6). Indeed, a low-frequency vibration determines a tissue deformation that can be assessed and quantified by imaging modalities like MR and ultrasound. From measurements of tissue elasticity, it is possible to calculate values of stiffness reconstructing the mechanical properties via inversion algorithms [66]. Thus, elastography might be applied to identify and monitor stiffness changes of human tissues in different disorders and may be useful to derive mechanical properties for the construction of finite element models.

MR elastography (MRE) has the advantage of providing a large and deep field of view. To date, MRE has been tested in some musculoskeletal settings with promising results. MRE seems to be feasible and reproducible in differentiating the nucleus and annulus regions of the intervertebral disc *in vivo* [67]. Further, MRE has demonstrated to detect mechanical property changes of the disc, with shear stiffness measurements having shown to highly correlate with intervertebral disc degeneration [68]. Preliminary phantom and *in vivo* studies showed the feasibility of this technique to assess mechanical and functional properties of skeletal muscles under passive (rest) conditions [69] and after activation with dynamic exercises [70]. This data might be used as a reference in future studies on muscle disorders in addition to other quantitative MR imaging biomarkers of muscle structure and function [71].

Ultrasound elastography is widely available, easy to perform, cheaper, and faster than MR. Strain elastography applies compressive forces and allows assessing tissue stiffness through a qualitative evaluation or ratios with neighbor tissues. Shear wave elastography, in turn, provides a quantitative assessment of shear wave propagation in tissues. According to the last clinical indications for musculoskeletal ultrasound by the European Society of Musculoskeletal Radiology, ultrasound elastography has now for Achilles tendinopathy an evidence level of B and an indication grade of 3, thereby being considered the first choice technique for this condition [72]. Indeed, it is well-known that tendon thickness, echotexture, and neovascularisation detected by B-mode and power-Doppler do not completely express the actual status of Achilles tendinopathy [73]. Ultrasound elastography has also been advocated as a potential complementary diagnostic tool to better characterise soft tissue masses [74, 75], although controversial results have been published with elastography having still no clear additional role to B-mode currently [76].

### dGEMRIC, T1rho, and T2 mapping

**Fig. 7:**
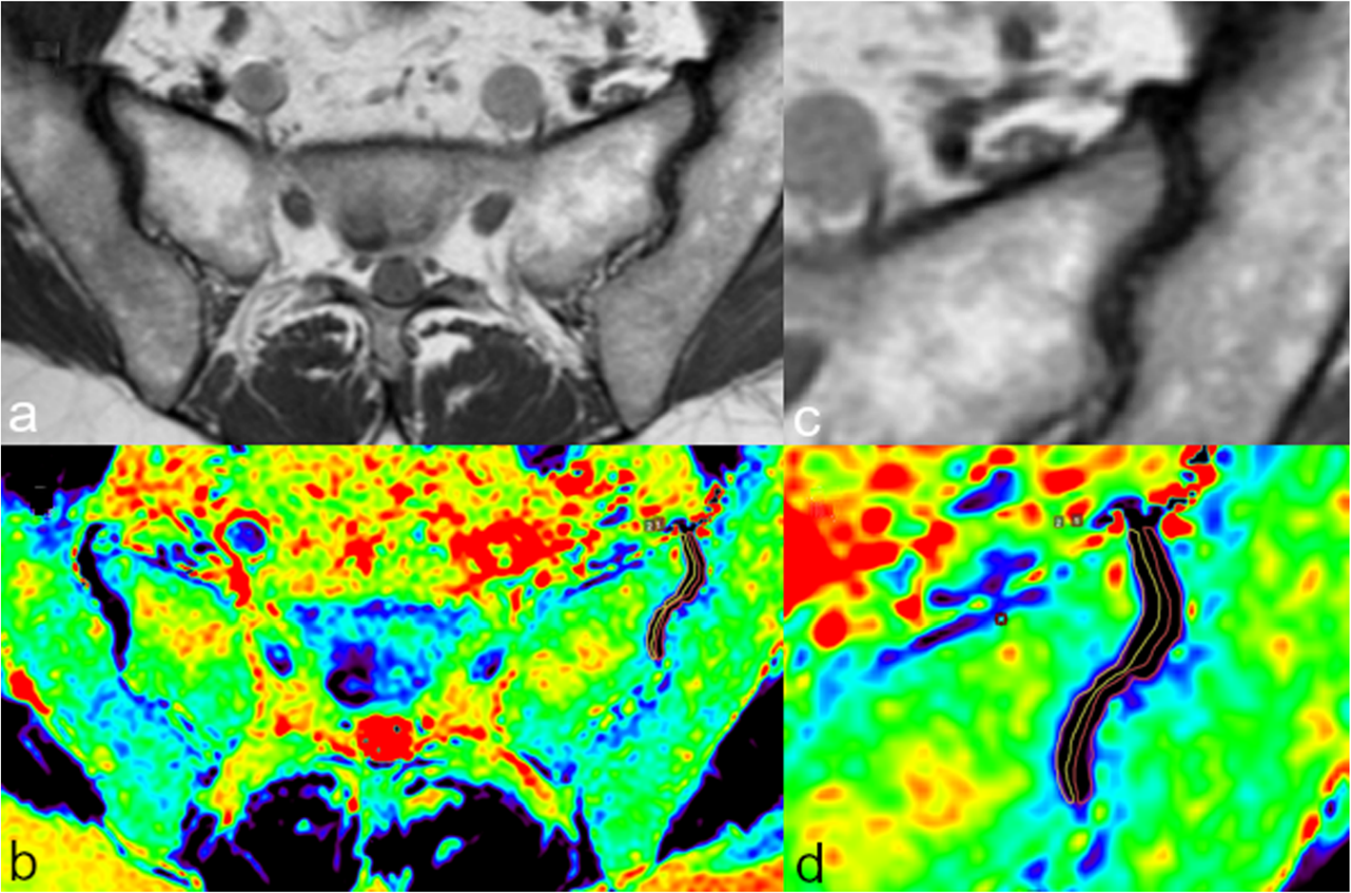
Magnetic resonance imaging of the sacroiliac joints of a 42-year-old female patient. Oblique axial T1-weighted turbo spin-echo image of both sacroiliac joints (**a**); in detail the left sacroiliac joint (**c**). The corresponding oblique axial T2 maps (**b**, **d**) show the ROIs manually drawn on both sacral and iliac articular side of the joint space of the left sacroiliac joint

MRI is the gold standard technique for the evaluation of cartilage disease, with fat-suppressed proton density and T2-weighted sequences being generally used for this purpose [77]. Nevertheless, standard MRI scans are not able to early detect cartilage and intervertebral disc ultrastructure changes. To date, a number of quantitative MR tools exist to identify biochemical information about cartilage health and subtle abnormalities of collagen fiber architecture, water, and proteoglycan content within the joint cartilage, including dGEMRIC, T1rho, and T2 mapping [78] (Fig. 7). Such quantitative information can be valuable for the development of finite element models, especially for multi-physical models which account for the presence of a fixed charge density, swelling phenomena, and a complex anisotropy deriving from the microarchitecture of the tissue of interest.

The dGEMRIC requires the intravenous injection of Gadolinium-based contrast agent that distributes throughout the cartilage. The contrast agent (positively charged) is repulsed from the glycosaminoglycans (negatively charged), thereby its distribution inversely correlates with glycosaminoglycans content [79]. Thus, T1 relaxation can be applied to indirectly assess the glycosaminoglycans content of the cartilage [80]. Nevertheless, the use of non-contrast functional MR sequences has been preferred over the last years especially in view of recent findings of Gadolinium accumulation in human tissues after multiple contrast-enhanced MR examinations [81].

T1rho allows to assess the interactions of water molecules with proteoglycans/glycosaminoglycans, since protons dissipate more energy when they are in proximity of these macromolecules [82]. Hence, proteoglycans/glycosaminoglycans depletion is associated with longer T1rho values. This makes T1rho highly sensitive for early changes of joint cartilage, as decrease content of these macromolecules precedes the disruption of collagen and the increase of water content [83]. The main issue of this sequence is the high specific absorption rate and the risk of tissue heating due to the power of radiofrequency waves.

T2 mapping results from the evaluation of T2 relaxation times of tissues after the acquisition of images of the same slice using multiple echoes (multi-echo sequence) [84, 85]. T2 mapping allows indirectly evaluating the collagen architecture of the cartilage. Indeed, the extracellular matrix of healthy cartilage traps the water molecules, leading to its typical low T2 signal intensity. When the collagen matrix breaks down, it becomes permeable to water with an increase of T2 relaxation times [86]. Of note, the main cons of T2 mapping are its susceptibility to the magic angle effect and the fact that proteoglycans/glycosaminoglycans depletion precedes the increase of water content related to extracellular matrix breaking down [87].

## Future perspectives and conclusions

The last years have seen the imposing rise of artificial intelligence and machine learning in many research and industrial field, including radiology. With the exception of automatic segmentation, these techniques did not gain a widespread use among the biomechanical community yet, in part due a rather conservative mindset and the mechanical engineering background of many researchers working in this field, in which computer science play a minor part. Nevertheless, papers in which artificial neural networks have been used to improve the accuracy of finite element models of the musculoskeletal system are starting to appear and show indeed great potential [88, 89]. Besides, finite element analysis may also benefit from the recent advances in artificial intelligence-based predictive models, which can guide and enhance the extraction of clinically relevant results from the numerical simulations by integrating the biomechanical results with predictions based on available medical knowledge and data. In summary, we believe that these disruptive technologies will have a major impact in the field of finite element analysis, and research efforts and funds should be dedicated to a better integration between FEA, artificial intelligence, and the large databases of clinical and radiological data which are necessary for the development of such models.

The paradigm of personalised or precision medicine, in which decisions and treatments are tailored on the individual patient with the aim of maximising benefits while limiting risks, is one of the major new frontiers in medical research. The paradigm is based on a high-resolution model of the individual patient, also known as “digital twin,” which covers anatomy, genetics, response to drugs, etc. In the musculoskeletal field, a personalised model needs to take into account biomechanical aspects, since the success of the treatments is inherently associated with biomechanical variables such as bone quality, body weight, and the individual anatomy in general. Personalised finite element models are therefore a key step for precision musculoskeletal medicine, and we expect that their importance will rise in the next future. As discussed in the previous paragraphs, whereas the current technologies allow for a satisfactory reconstruction of the individual anatomy based on medical images and research efforts are mostly dedicated to reduce the necessary workload, obtaining personalised material properties remains challenging, and further research in this field is warranted. Personalised loading and boundary conditions, which are also necessary for patient-specific biomechanical modeling and have not been discussed in this paper since they are not related with medical imaging, are also a challenge and indeed a field of active research.

Another direction of expected developments as regards the commercialisation of imaging software that allows for obtaining mechanical information more and more accessible and easy to use, also thanks to a closer collaboration with industrial partners. Implementing the use of these tools in clinical practice will further facilitate the transition from conventional to quantitative and personalised imaging.

In conclusion, we presented a brief overview of the techniques used for segmenting medical images, building a finite element mesh, and assessing the mechanical properties of biological tissues from imaging data, with the aim of developing finite element models for the biomechanical simulation of the musculoskeletal system. Both consolidated methods, many of which have been used for decades, and recent advances such as those based on artificial intelligence have been described. We believe that the recent and forthcoming innovations in the latter field will have a major impact on numerical analysis, fostering the use of patient-specific biomechanical modeling in personalised medicine approaches.

## Data Availability

No datasets are associated with this paper.

## Abbreviations

2D: Two-dimensional
3D: Three-dimensional
ANN: Artificial neural network
ASD: Asymmetric surface distance
BMD: Bone mineral density
CNN: Convolutional neural network
CT: Computed tomography
dGEMRIC: Delayed gadolinium-enhanced MRI of cartilage
DXA: Dual energy x-ray absorptiometry
FEA: Finite element analysis
HR-QCT: High-resolution quantitative computed tomography
MRE: Magnetic resonance elastography
MRI: Magnetic resonance imaging
QCT: Quantitative computed tomography
